# Subchronic exposure to phytoestrogens alone and in combination with diethylstilbestrol - pituitary tumor induction in Fischer 344 rats

**DOI:** 10.1186/1743-7075-7-40

**Published:** 2010-05-10

**Authors:** Yow-Jiun Jeng, Mikhail Kochukov, Dhananjaya Nauduri, Bhupendra S Kaphalia, Cheryl S Watson

**Affiliations:** 1 Department of Biochemistry and Molecular Biology, University of Texas Medical Branch, Galveston, Texas, USA; 2 Department of Pathology, University of Texas Medical Branch, Galveston, Texas, USA

## Abstract

**Background:**

Subchronic administration of the potent pharmaceutical estrogen diethylstilbestrol (DES) to female Fischer 344 (F344) rats induces growth of large, hemorrhagic pituitaries that progress to tumors. Phytoestrogens (dietary plant estrogens) are hypothesized to be potential tumor inhibitors in tissues prone to estrogen-induced cancers, and have been suggested as "safer" estrogen replacements. However, it is unknown if they might themselves establish or exacerbate the growth of estrogen-responsive cancers, such as in pituitary.

**Methods:**

We implanted rats with silastic capsules containing 5 mg of four different phytoestrogens - either coumestrol, daidzein, genistein, or *trans*-resveratrol, in the presence or absence of DES. We examined pituitary and other organ weights, blood levels of prolactin (PRL) and growth hormone (GH), body weights, and pituitary tissue histology.

**Results:**

Blood level measurements of the administered phytoestrogens confirmed successful exposure of the animals to high levels of these compounds. By themselves, no phytoestrogen increased pituitary weights or serum PRL levels after 10 weeks of treatment. DES, genistein, and resveratrol increased GH levels during this time. Phytoestrogens neither changed any wet organ weight (uterus, ovary, cervix, liver, and kidney) after 10 weeks of treatment, nor reversed the adverse effects of DES on pituitaries, GH and PRL levels, or body weight gain after 8 weeks of co-treatment. However, they did reverse the DES-induced weight increase on the ovary and cervix. Morphometric examination of pituitaries revealed that treatment with DES, either alone or in combination with phytoestrogens, caused gross structural changes that included decreases in tissue cell density, increases in vascularity, and multiple hemorrhagic areas. DES, especially in combination with phytoestrogens, caused the development of larger and more heterogeneous nuclear sizes in pituitary.

**Conclusions:**

High levels of phytoestrogens by themselves did not cause pituitary precancerous growth or change weights of other estrogen-sensitive organs, though when combined with DES, they counteracted the growth effects of DES on reproductive organs. In the pituitary, phytoestrogens did not reverse the effects of DES, but they did increase the sizes and size heterogeneity of nuclei. Therefore, phytoestrogens may oppose some but not all estrogen-responsive tissue abnormalities caused by DES overstimulation, and appear to exacerbate DES-induced nuclear changes.

## Background

Steroid hormones such as the dominant physiologic estrogen, estradiol (E_2_) have many effects on pituitary function, including regulation of most pituitary hormones and proliferation of several pituitary cell types [1,2]. The Fischer 344 (F344) rat has long been used as a model for investigating growth control of estrogen-responsive tissues (especially those prone to estrogen-induced tumors), by various estrogens, and related biological processes such as angiogenesis [3,4]. When female F344 rats are chronically treated with E_2 _or the pharmaceutical estrogen diethylstilbestrol (DES), their pituitaries grow 10 to 20 times normal size and sometimes form a tumor by 10 weeks [5,6].

Estrogens can increase the expression levels of basic fibroblast growth factor and pituitary tumor transforming gene products in F344 animals [1,2], leading to prolactinoma development, vascularization, and increases in cell number, which have been identified as quantifiable genetic traits [5,7].

The peptide hormone prolactin (PRL) is expressed in the pituitary of all mammals, and its major function in females is the stimulation of milk production by the mammary gland. Additional known functions include modulation of other aspects of reproduction, osmoregulation, growth, metabolism, and migratory and maternal behaviors [2]. Hyperprolactinomas release highly elevated plasma PRL levels leading to reproductive dysfunction in both males and females [8,9]. There is also a tight correlation between E_2 _levels and growth hormone (GH) secretion by the pituitary. Serum GH responds to changes in E_2 _levels during different life stages in women [10,11] and regulates body growth and composition, metabolism, bone density and pubertal development in both sexes [12].

Phytoestrogens are plant-derived compounds that structurally and functionally mimic mammalian endogenous estrogens. These compounds have been considered candidate therapeutic or prevention agents for such diseases as reproductive system cancers, heart disease, menopausal symptoms, and osteoporosis - essentially mimicking the health benefits thought to be characteristic of endogenous estrogens, while counteracting the hazards [13,14]. Considering the numerous beneficial effects of estrogens, it is not surprising that phytoestrogens are considered possible complementary or alternative medicine treatments. However, some estrogens are associated with detrimental effects over life-long exposures. For instance, cumulative high exposures to endogenous, therapeutic, or environmental estrogens have been implicated in diseases such as breast cancer [2,15-17]. Recently, breast cancer incidence in a large human population was noted to be inversely correlated to the consumption of soy phytoestrogens in the diet [18]. Therefore, we need to carefully examine the beneficial vs. the detrimental biological effects of phytoestrogens in animal studies.

Isoflavones, including the components of soy bean-derived foods such as genistein and daidzein, are some of the most studied phytoestrogens. Part of the original reasoning behind proposing potential health benefits of phytoestrogens stemmed from the fact that those consuming "Asian diets" high in soy isoflavones seem to be less vulnerable to the diseases of both estrogen overexposure (cancers) and estrogen underexposure (osteoporosis, hot flashes, heart disease, depression, etc.). These benefits are thought to be diet-related rather than genetic, because when Asians move to Western countries and adopt their diets, their incidences of these diseases become similar to Westerners [19,20]. Coumestrol which is supplied by foods such as alfalfa sprouts, or is transmitted to the diet via red clover consumption by livestock, is also thought to have these beneficial effects. Resveratrol consumption, also speculated to be beneficial, could explain why populations that daily consume red wine (which contains high levels of resveratrol) benefit by having lower levels of diseases thought to be associated with estrogen deficits (eg. heart disease).

The different activities and the bioavailability of phytoestrogens vary depending on factors such as the route of administration, dosage, individual metabolism, co-ingestion of other substances, and phytoestrogen levels present in intake foods [21,22]. For example, in Japanese men and women consuming a traditional diet, the plasma isoflavone concentrations can be as high as 0.2 to 1 μM. In Europe and North America, plasma concentrations for isoflavones are between 0.005 and 0.4 μM [22-24]. The blood concentrations for coumesterol can be from 0.01 (reported as an average from food intake, Malaysia [25]) to 0.5 μM (resulting from taking dietary supplements [24]). Resveratrol, a compound which has very low bioavailability and is rapidly metabolized in humans [26,27], has serum concentrations (for the compound and its metabolites) as high as 2 μM [28]. In the present studies, these four phytoestrogens were provided via sustained slow release directly to the circulation, bypassing gut microflora and hepatic first pass metabolism, which can have a major impact on the biological potency of phytoestrogens. We examined estrogenic effects on size and architecture of multiple organs (both reproductive and not), and on body weights, in female F344 rats. In particular we focused on the size, structure, cellular composition, and function of the pituitaries, as this tissue is used to monitor carcinogenic estrogenic effects in this animal model. Our studies investigate whether phytoestrogens mimic, inhibit, or exacerbate the known effects of DES.

## Methods

Cholesterol, flavone, DES, *trans*-resveratrol, coumesterol, daidzein, and genistein were obtained from Fluka (Milwaukee, WI) or Sigma Chemical Co. (St. Louis, MO). The high purity grade solvents and silica gel were purchased from Fisher Scientific (Pittsburgh, PA).

### Animals and hormone treatment

There are several issues which often arise in dietary treatment regimens for phytoestrogens. The rationale for our protocol limits animal use and manipulations, while still addressing important issues of comparisons of phytoestrogen modulation of estrogenic carcinogenesis. We used a high level of phytoestrogen exposure to determine if any harm could be caused by this exposure. Since these doses of phytoestrogens far exceed that which could be delivered by the animal feed (demonstrated by the blood levels of free compounds in our control animals being undetectable), we thought it unnecessary to feed a specialized diet to eliminate such a comparatively negligible source of phytoestrogens. Because we did not know whether simultaneous phytoestrogen exposure would inhibit or exacerbate tumor development, we used a sub-end point tumor development assessment time in animals receiving DES in combination with phytoestrogens. Were phytoestrogens to exacerbate tumor growth, then the animals might not survive for the entire 10 week induction period. We chose an 8 week endpoint which had been shown to still generate DES-induced growth effects, but to a less developed stage [29]. On the other hand, if any phytoestrogens alone were to have less of an effect on pituitary growth than DES, we reasoned that these effects would be difficult to observe at shorter exposure times. Therefore, we used the full 10 week time point for this outcome. Because we were using very high doses of DES (tumor induction levels) and phytoestrogens, the reproductive cycles of these animals would be overwhelmed with the effects of administered estrogens that have been shown to disrupt estrous cycles [30,31]. Also, the high doses used in these studies rendered the contribution of endogenous estrogens very minor, so we did not ovariectomize the test animals.

Female F344 rats (21 days old) were obtained from Harlan Sprague-Dawley (Indianapolis, IN) and housed (five rats per cage) in a controlled environment (light on, 0500-1900 hours, 22°C, and 50% humidity) with free access to water and food (Prolab RMH 2500 LabDiet, Richmond, IN). For implanting hormone-containing capsules, rats were anesthetized, followed by 5 mm neck incisions and subcutaneous placement of pieces of silastic tubing (Allied Biomedical, Paso Robles, CA). Animals were treated for 10 weeks with implants containing 5 mg of cholesterol (negative control), DES (positive control), coumesterol, daidzein, genistein, or *trans*-resveratrol (Sigma, St. Louis, MO). To investigate whether phytoestrogens inhibit or exacerbate the known effects of DES exposure in this paradigm (growth of pituitary and pituitary tumors), another set of animals received subcutaneous silastic implants containing 5 mg DES along with 5 mg of either cholesterol, coumesterol, daidzein, genistein, or *trans*-resveratrol for 8 weeks. There were 10 rats per group and animals were weighed weekly. Blood was collected from each animal into centrifuge tubes that contained no anti-coagulant at 4 weeks and at sacrifice for analysis of PRL and GH levels. To confirm successful release of phytohormones and DES by the implants, the collected blood from rats treated with single compounds was also assayed for free phytoestrogens or DES. Plasma was separated according to standard protocol and the samples aliquoted and stored at -80°C until analysis. Pituitaries, estrogen-sensitive reproductive organs (cervices, ovaries, uteri) and non-reproductive organs (kidneys and livers) were carefully dissected from surrounding tissues and weighed. Pituitaries were fixed in 4% PBS-buffered paraformaldehyde, followed by imbedding, sectioning (5 μm) and staining with hematoxylin and eosin [32]. This use of animals was approved by the Institutional Animal Care and Use Committee at UTMB.

### Extraction and analysis of phytoestrogens in the plasma

Flavone (1 ng, internal standard) was added to the rat plasma (250 μL) and incubated at 37°C for 30 min followed by an addition of 0.1 ml acetic acid and 2 mL methanol:diethylether (3:1, v/v). The contents were mixed well and sonicated for 2 min, then centrifuged at 600 × g for 15 minutes. Supernatants were transferred into clean glass vials and solvent was evaporated under a stream of nitrogen at 40°C. The residue was redissolved in 1 ml n-heptane and subjected to silica gel solid-phase extraction. The silica gel column was washed with 3 mL methanol followed by 3 mL n-heptane. The sample was loaded onto the silica gel column and then washed with 10 mL n-heptane. Elution was done by adding 5 mL methanol. The eluate was evaporated under nitrogen and redissolved in 200 μL of mobile phase A (23:24:53 acetonitrile: methanol: water) and analyzed by high performance liquid chromatography (HPLC).

Analysis of individual phytoestrogens was carried out on a Beckman Coulter System Gold (Pump module 125, PDA Detector 168, and manual injector). Data acquisition and post-run analysis were performed using 32Karat v7.0 combined with Gemini ODS [length 250 mm, particle size 5 μm, I.D. 4.6 mm, Phenomenex (Torrance, CA)]. Elution was done by acetonitrile: methanol: water (23:24:53, v/v) containing 0.01% trifluoro acetic acid flowing at 0.7 ml/min under isocratic conditions. The detector was set at 254 nm for DES, coumesterol daidzein, and genistein [33] and 306 nm for *trans*-resveratrol [34]. A minimum of 5 pmol of each compound was detectable by the method used in this study. Recovery of phytoestrogens was found to be > 90%. The data were corrected for the percent recovery.

### Assay of serum PRL and GH

The serum levels of PRL and GH were measured using an enzyme immunoassay kit from Alpco (Windham, NH) and Millipore (Billerica, MA) respectively, according to the manufacturer's instructions.

### Nuclear morphometry of the pituitary

Representative sections were analyzed using a Nikon Eclipse E800-UIC upright microscope equipped with a Nikon digital DXM 1200 color CCD camera and PL FL 10× objective (N.A. 0.3) controlled by ACT-1 acquisition software (Nikon, Melville, NY). Representative images (2-4) were acquired from each rat anterior pituitary. Acquired digital images were processed with Metamorph 7.0 software (Molecular Imaging, Downingtown, PA) using manual outlining of nuclear images for subsequent measurement of nuclear areas. Nuclei (80-200) were randomly selected from each image and measured. Results were averaged to obtain the mean nuclear size for each pituitary and then combined to plot a histogram. To compare different treatments, the average of the mean nuclear size from each individual animal's tissue was used.

### Statistics

Data from the morphometrics analysis of pituitary tissue, organ weights, serum PRL and GH levels, and serum concentrations of phytoestrogens were analyzed by one-way analysis of variance (ANOVA) followed by multiple comparisons versus control group (Holm-Sidak method). The Sigma Stat 3 program (Systat Software, Inc.) was used for all statistical analysis, and significance was accepted at p < 0.05.

## Results

### Serum levels of phytoestrogens after 4 and 10 weeks of treatment

To show the effectiveness of our silastic implant delivery system, we measured serum levels for DES and all phytoestrogens in F344 animals after 4 and 10 weeks of treatment (Table 1). All compounds were readily detectable in serum at both time points, and were near the higher end of those values obtained via dietary exposure reported for both rodents and humans. The serum levels of DES, coumesterol, and genistein were higher in blood collected after 10 weeks compared to 4 weeks of treatment, but resveratrol levels were highest after only 4 weeks. Neither daidzein nor *trans*-resveratrol treatments resulted in further increases in the amounts of the compounds in blood at 10 weeks. No detectable levels of these phytoestrogens were found in control animals whose implants contained cholesterol.

**Table 1:** Serum concentrations of phytoestrogens

Treatment	4^th ^week	10^th ^week	Reported rat serum concentrations (ng/ml)
DES	20 ± 5 ng/ml	353 ± 60 ng/ml	For comparison, serum E_2 _levels in cycling
	(0.07 ± 0.02 μM)	(1.3 ± 0.2 μM)	F344 rats is 1-16 pg/ml (4-59 pM) [54]
Coumesterol	182 ± 18 ng/ml	590 ± 83 ng/ml	ND-10 ng/ml (0.04 μM) [52]
	(0.68 ± 0.07 μM)	(2.2 ± 0.31 μM)	
Daidzein	30 ± 2 ng/ml	43 ± 5 ng/ml	ND-13 ng/ml (0.05 μM) [52]
	(0.12 ± 0.07 μM)	(0.17 ± 0.02 μM)	
Genistein	14 ± 5 ng/ml	117 ± 12 ng/ml	ND-28 ng/ml (0.10 μM) [52]
	(0.05 ± 0.02 μM)	(0.43 ± 0.04 μM)	
*T*rans-resveratrol	58 ± 15 ng/ml	49 ± 9 ng/ml	ND [27,55]
	(0.25 ± 0.06 μM)	(0.21 ± 0.04 μM)	

Free phytoestrogens in blood from animals with implants for 4 or 10 weeks (n = 10 animals per group) are shown in both ng/ml and molar concentrations. Five blood samples from each group were analyzed. The control animals (with cholesterol implants) had below detectable levels for all measured compounds. The last column lists referenced phytoestrogen levels in rat serum from untreated rats. ND = nondetectable.

### Effects of phytoestrogens on pituitary weights, PRL levels, GH levels, and body weights (Figure 1)

**Figure 1:**
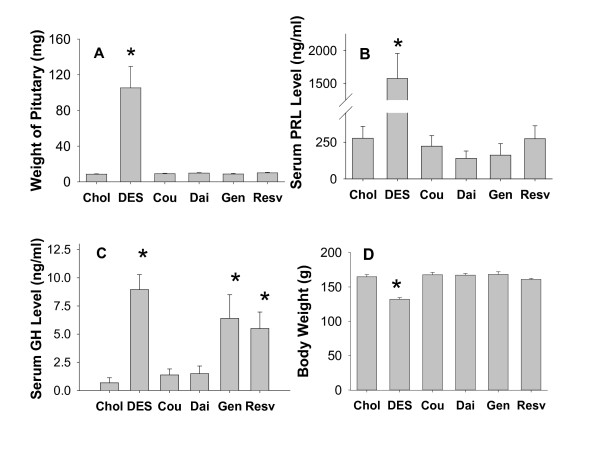
**Pituitary weight and functions, and body weight in animals treated with DES or phytoestrogens**. A. wet pituitary weights; B. serum PRL levels; C. serum GH levels; and D. body weights of animals treated with DES (diethylstibesterol), Cou (coumesterol), Dai (daidzein), Gen (genistein), or Resv (*trans*-resveratrol) for 10 weeks. * = p < 0.05 compared to control animals treated with cholesterol (Chol) only. n = 10 animals for each group.

Pituitaries were greatly increased in size after 10 weeks treatment with DES. As previously reported for this experimental model, the weight increase was ~10-fold compared to control animals. In DES-treated animals, the serum PRL levels were elevated ~6-fold, and the GH levels were elevated ~9-fold. The body weights of DES-treated animals were significantly lower than control animals. None of the four different phytoestrogen treatments altered the pituitary weights, PRL levels, or body weights of these F344 rats. However, among the phytoestrogens, both genistein and resveratrol caused a 6-fold increase in GH levels.

### Effects of phytoestrogens on reproductive and nonreproductive organ weights (Figure 2)

**Figure 2:**
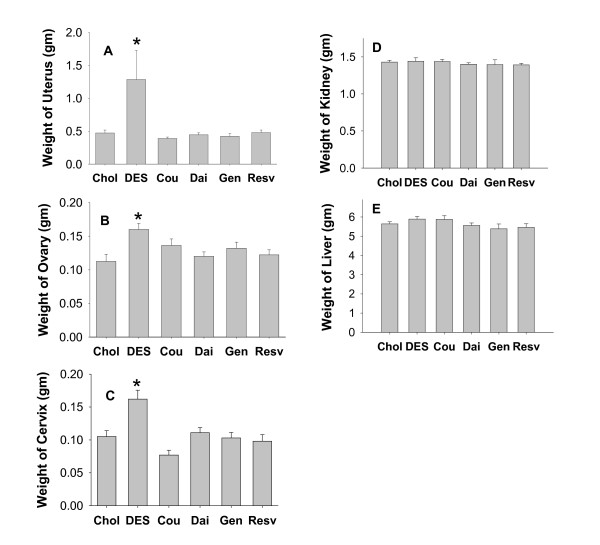
**DES- or phytoestrogen-induced wet organ weights**. The wet weight of organs from animals treated with DES, Cou, Dai, Gen, and Resv for 10 weeks including A. uterus; B. ovary; C. cervix; D. kidney; and E. liver. The abbreviations are as defined in the legend of Figure 1. * = p < 0.05 compared to control (Chol) animals. n = 10 animals for each group.

We also examined the wet weight of several organs from animals exposed to these various estrogen implants for 10 weeks. DES treatment increased wet weights of reproductive organs (ovaries, uteri, and cervices) at 10 weeks, as expected. However, none of the phytoestrogens had any significant effects on reproductive organs (Figure 2A-C). The weights of livers and kidneys were not significantly affected by either DES or any of the tested phytoestrogens (Figure 2D-E).

### Effects of combinations of DES and phytoestrogens on pituitary weights, PRL levels, GH levels, and body weights (Figure 3)

**Figure 3:**
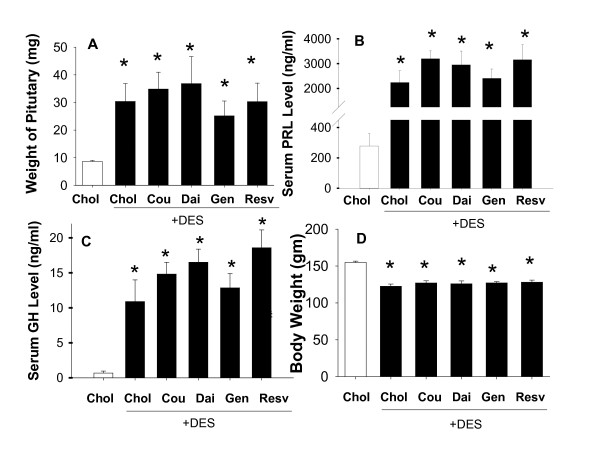
**Pituitary weight and functions, and body weight in animals treated with DES + phytoestrogens**. A. The wet weights of pituitaries; B. serum PRL levels; C. serum GH levels; and D. body weight, from animals treated without (open bar) or with DES (dark bars) in combination with Chol (control), Cou, Dai, Gen, and Resv for 8 weeks. The abbreviations are as defined in the legend of Figure 1. * = p < 0.05 compared to control (Chol) animals without DES treatment. n = 10 animals for each group. There were no statistically significant differences between animals treated with DES alone, compared to DES + phytoestrogens.

Pituitary weights increased significantly (~3 fold) after 8 weeks of treatment with DES, as expected for this nonlinear tumor development model system [3], whether or not the phytoestrogens were co-administered; therefore, no phytoestrogen treatment significantly changed the size progression of DES-treated pituitaries, in either a positive or negative direction. Serum PRL and GH levels were also significantly elevated in animals treated with DES alone; again, co-treatment with phytoestrogens did not alter this elevation. Essentially, the pituitary weight effects were reflected in the similar increases in PRL levels. The body weights were significantly (25%) lower in DES-treated animals, regardless of whether it was administered with or without any of the phytoestrogens. Therefore, phytoestrogen treatment did not significantly change the estrogenic effects of DES.

### Effects of combinations of DES and phytoestrogens on reproductive and nonreproductive organ weights (Figure 4)

**Figure 4:**
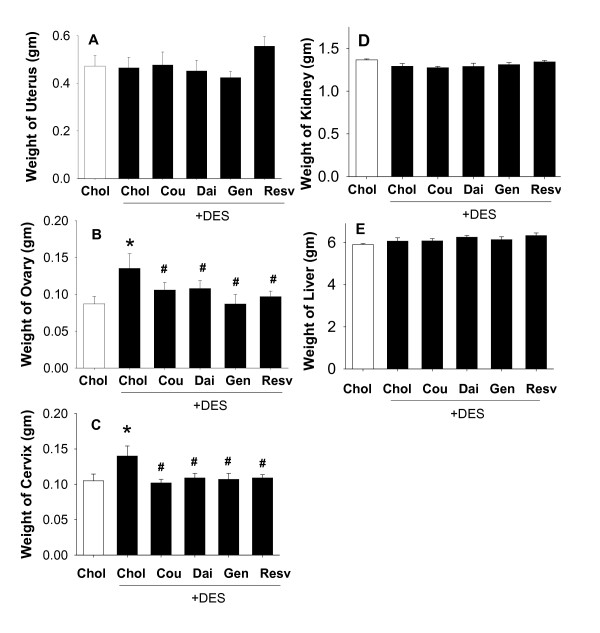
**Wet organ weights as affected by DES treatment in combination with phytoestrogens**. The weight of reproductive organs including: A. uterus; B. ovary; C. cervix; and non-reproductive organs: D. kidney; and E. liver, from animals treated without (open bar) or with DES (dark bars) in combination with Chol (control), Cou, Dai, Gen, and Resv for 8 weeks. The abbreviations are as defined in the legend of Figure 1. * = p < 0.05 compared to control (Chol) animals without DES treatment. # = p < 0.05 vs. DES alone. n = 10 animals for each group.

We also examined the reproductive organs of F344 rats co-treated with DES and different phytoestrogens (Figure 4A-C). Interestingly, DES did not increase the size of the uterus in these studies of only 8 weeks of DES exposure (even though the 10 week exposure did cause this effect, see Figure 2). Yet in the same animals, ovary and cervix weights were increased by this 8 week treatment, so it is apparent that the animals were exposed to an effective dose of DES. All phytoestrogens attenuated the effects of DES size gain in ovaries and cervices. The weights of livers and kidneys were not affected by DES alone or in combination with any of the tested phytoestrogens (Figure 4D-E), similar to those results obtained at 10 weeks with single compounds (see Figure 2).

### Nuclear changes in anterior pituitary induced by phytoestrogens and DES

**Figure 5:**
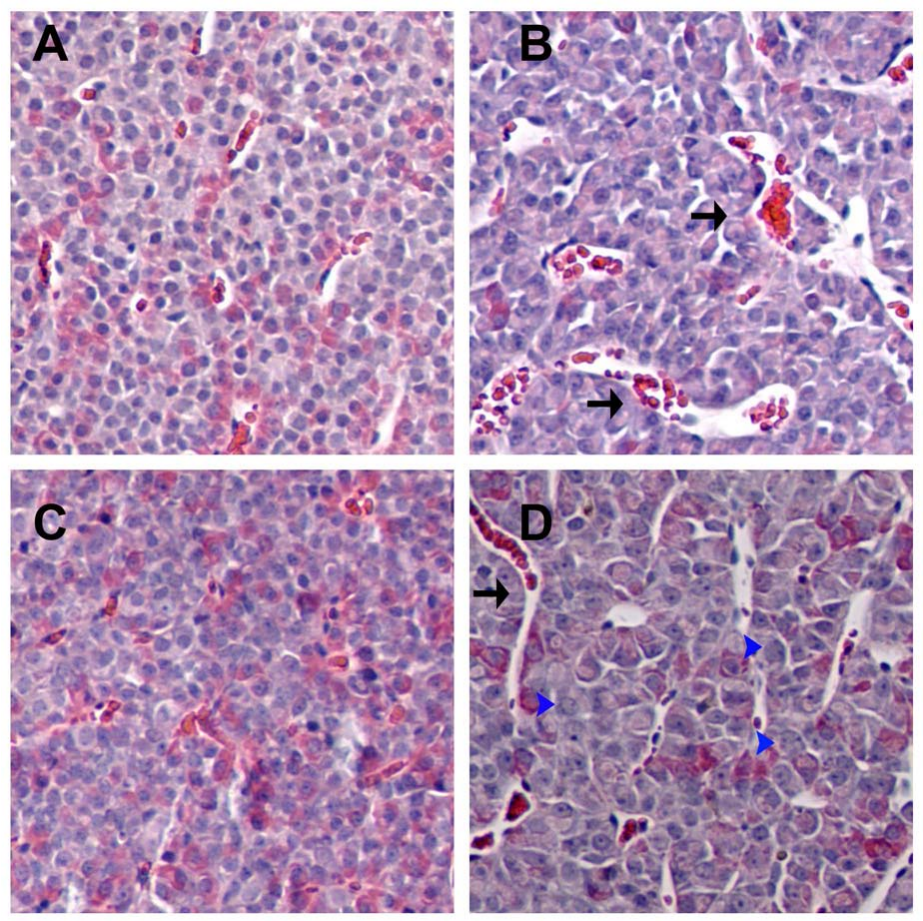
**Representative stained pituitary tissues from animals treated with DES, daidzein, or the combination**: The hematoxylin and eosin staining of pituitary tissues were recorded at 400× magnification from animals treated with A. cholesterol; B. DES; C. daidzein; or D. daidzein + DES. Note the large vascular and hemorrhagic areas (black arrows in B and D) resulting from DES treatments, regardless of whether daidzein (or other phytoestrogens) were administered in combination. Also note the larger nuclear size in animals treated with daidzein + DES (blue arrowheads in D). Combination treatments with DES plus other phytoestrogens gave similar results.

**Figure 6:**
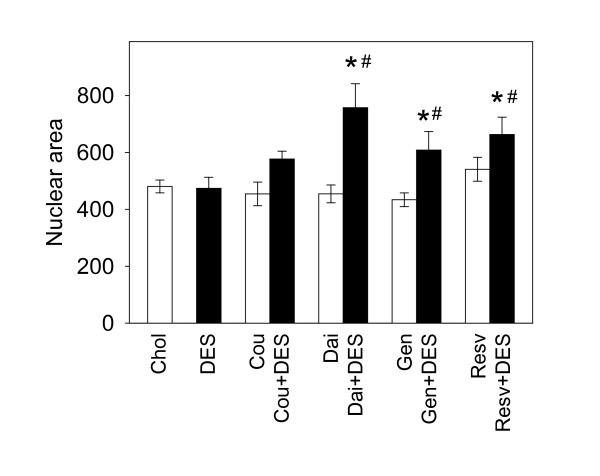
**Nuclear morphometric changes in rat anterior pituitaries induced by phytoestrogens, DES, or combination treatments**. Average nuclear areas were estimated as described in methods (n = 4-8 animals for each treatment group), * = p < 0.05 vs. control (Chol), # = p < 0.05 vs. DES alone.

**Figure 7:**
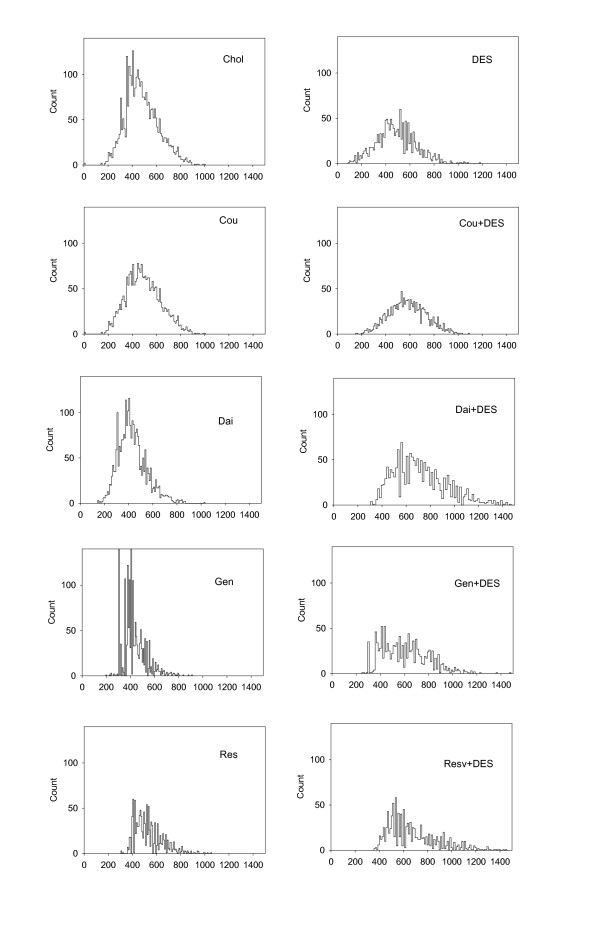
**Nuclear size histogram of pituitary cells**: Individual nuclei (n = 1200-3000 for each group) measurements for anterior pituitaries were plotted as the 100-bin histogram, reflecting their variability and distribution by size. The Y axis shows the number of nuclei counted and X-axis shows the ranked nuclear size. See table 2 for the percentages of cells with large nuclear sizes (> 800).

DES treatment, either alone or in combination with phytoestrogens, caused gross structural changes in rat anterior pituitaries that included decrease in cell density in the tissue, increase in vascularity, and multiple areas of hemorrhages (representative pictures shown in Figure 5). To investigate whether there were any other quantifiable microscopic changes, we also examined the nuclear sizes of pituitary cells from each animal (Figure 6) using software-assisted micromorphotometric techniques. Although none of the phytoestrogens alone produced significant changes in this parameter, combinations of daidzein, genistein, or resveratrol with DES treatment resulted in significantly larger nuclear areas compared to control or DES alone treatment groups. [Although the coumestrol + DES treatment group was not significantly different from treatment with either of the single compounds alone, the differences approached significance (P = 0.139 compared to DES and 0.129 for coumestrol)]. As seen in the histogram of nuclear sizes (Figure 7), this observed increase in average nuclear area was accompanied by a marked increase in nuclear size polymorphism (multiple peaks in the nuclear size histogram) as well as multiple giant nuclei, especially in the DES + phytoestrogens treatment groups. In each case, treatment with DES plus a phytoestrogen broadened the profile in the direction of larger and more heterogenously-sized nuclei. This is quantified in Table 2 which shows the % of cells that have a nuclear area of a > 800 value for ranked nuclear size.

**Table 2:** Combinations of DES and phytoestrogens increase the % of cells that have large nuclear size.

Treatment	% cells > 800 Without DES	% cells > 800 With DES
Cholesterol	1.67	3.71
Coumesterol	1.97	8.94
Daidzein	0.80	30.17
Genistein	0.24	16.35
*Trans*-resveratrol	3.13	23.49

The percent of cells with nuclei areas above the 800 value for ranked nuclear size (see Figure. 7) is shown.

## Discussion

These studies examined organ size and functional responses to several phytoestrogens, in comparison to the actions of a well-studied pharmaceutical estrogen-induced model for carcinogenicity. The stimulatory effects of DES treatment on pituitary function were robust (as expected for this well established model) in these young (21 day old) female F344 rats treated with estrogens for ~2 months. Large impacts on pituitary functions and reproductive maturation was expected in animals whose human life-span equivalency approximated the beginning of puberty. DES treatment increased the size, as well as caused structural changes, in the pituitary. However, phytoestrogen treatments, even at these high concentrations, neither caused the same effects, nor attenuated the effects of DES. DES treatment also increased serum PRL and GH levels, and two phytoestrogens, genistein and resveratrol, also caused significant increases in serum GH levels. Genistein was previously reported to increase GH in ewes and rats [35,36]. These kinds of effects may be related to estrogenic effects on stature in both boys and girls [37]. Although daidzein is very similar in chemical structure to genistein, it did not increase the serum GH levels. This may be due to the lower serum daidzein levels at 10 weeks compared to genistein, or to the broader signaling capabilities of genistein (for instance to also inhibit tyrosine kinases [36]). These effects are clearly separable from the effects on pituitary weight in our study, as no phytoestrogens were able to change the pituitary weights. Known effects of GH and PRL do not explain the phytoestrogen-induced reversal of DES-induced growth on the cervix and ovary that we observed. However, as we have shown previously in pituitary tumor cell lines, some of the abilities of phytoestrogens to change tissue growth patterns could be related to their capacity to differentially activate caspases and several mitogen-activated protein kinases [38,39] via nongenomic signaling mechanisms.

The average nuclear size in pituitary cells (which reflects their functional state) is known to be changed due to perturbations in hormonal status [40-42]. Although the average nuclear size from pituitaries of DES-treated or phytoestrogen-treated animals showed no difference compared to cholesterol-treated control animals, the observed changes in nuclear size heterogeneity with DES (and especially with DES in combination with all phytoestrogens), suggested that the functions of pituitary cells had been altered. High serum PRL and GH levels as well as pituitary size increases in animals treated with DES suggest that nuclear heterogeneity can be a marker for peptide hormone production and/or growth in the pituitary, though elevated GH levels caused by genistein did not correlate with such nuclear changes. It is interesting that phytoestrogens increased nuclear area only when their actions were superimposed on DES effects, and that the corresponding serum PRL and GH levels did not reflect any additional changes due to these combinations. Increases in nuclear size measured by tissue morphometry have been previously correlated to high risk pre-invasive breast lesions [43], and pituitary tumors are known to have large pleiomorphic nuclei [44]. Estrogens have also been shown recently to alter nuclear morphology related to MAPK activation and changes in cell division machinery [45]. Though the nuclear size changes we observed are not directly connected to a known health risk, they could signal some tissue growth instabilities brought on by excessive and diverse estrogenic exposure which would require more exposure time to manifest their effects. However, the levels of exposure that we examined were very high and were intended to demonstrate actions in an experimental situation where DES was known to cause pituitary tumorigenesis over a short time period.

Our use of this animal model for carcinogenesis demonstrated the expected estrogenic overstimulation by DES on the reproductive organs of female F344 rats. Others have reported that estradiol or DES treatment increases uterine wet weight, epithelial thickness, loose density stroma, and development of more uterine glands [46]. They also regulate cervical epithelial cell proliferation [47] and increases ovarian wet weight [48], including in F344 rats. However, phytoestrogens by themselves had none of these effects at the organ weight level, though they were able to suppress the weight gain effects of DES in ovaries and cervices after 8 weeks of co-treatment. So where reproductive organs show estrogenic effects, phytoestrogens may be an effective foil.

Only DES caused lower body weights in our study, which could not be reversed by phytoestrogens. Sex hormones are known to regulate rat body weights [49]; possible mechanisms include decreased metabolism and decrease lipoprotein lipase activity in adipose tissues [50]. Other studies have suggested that estrogens, especially at high concentrations, can cause nausea, indirectly contributing to poor eating and weight loss, which could contribute to the prevalence of anorexia in adolescent girls [51]. None of the high dose phytoestrogens in our study caused weight loss effects similar to DES, at least not for such subchronic exposures.

There is a concern that phytoestrogens present in animal feeds may affect experimental outcomes and also cause phytoestrogen exposures to humans consuming livestock fed on such diets [19,21]. Blood phytoestrogen content in our control animals was not detectable, and if present, negligible compared to the high concentrations delivered by our implants. The fact that the levels of these compounds were undetectable in the cholesterol-fed control animals using our sensitive extraction and assay methods, correlates with the amounts attributable to animal feed previously studied [52].

The increase in serum levels of coumesterol, daidzein, and genistein from 4 to 10 weeks suggests bioaccumulation, as has been suggested by previous studies [53]. Though factors such as species, age, developmental status, gender, diet, dose, route of administration, and metabolism all influence the ultimate phytoestrogen exposure, limiting the effectiveness of comparisons between studies in both rodents and humans, the levels of serum phytoestrogens that we report are comparable to high human levels. It appears that even such high doses of phytoestrogens did not cause effects in the organs or hormonal systems that we examined, except to alter some microscopic tissue architecture when in combination with a carcinogenic estrogen (DES). However, with some exceptions on reproductive organs, neither did they counteract the effects of this known potent estrogenic carcinogen.

## Conclusions

Our studies demonstrated that phytoestrogens by themselves, even in very high subchronic doses (that could match, however, accumulations in human blood via dietary intake [22-25,28]), did not cause the growth of pituitaries to sizes associated with tumor development, nor did they cause the growth of other reproductive, nonreproductive, or xenoestrogen-processing metabolic organs (liver or kidney). Though the phytoestrogens were unable to attenuate the effects of DES treatment on PRL and GH levels, pituitary weight, and body weight, they were able to reverse the DES-induced reproductive organ weight gains. So, it is these effects which may contribute to any protection such estrogens may give from endogenous or environmental estrogen overexposure. Concerns that have been raised about safety of these compounds as dietary supplements are not supported by many of our results, though the carcinogenic estrogen + phytoestrogen effects that we showed on nuclear size and heterogeneity should be studied further.

## Competing interests

The authors declare that they have no competing interests.

## Authors' contributions

DN and BSK analyzed serum phytoestrogens level in these studies, MK performed the morphometric analysis of pituitaries, and YJJ carried out the rest of studies. YJJ and CSW both participated in the design of the study and statistical analyses. All authors read and approved the final manuscript.
